# A Closer Look at the Genomic Variation of Geographically Diverse *Mycobacterium abscessus* Clones That Cause Human Infection and Disease

**DOI:** 10.3389/fmicb.2018.02988

**Published:** 2018-12-05

**Authors:** Rebecca M. Davidson

**Affiliations:** Center for Genes, Environment and Health, National Jewish Health, Denver, CO, United States

**Keywords:** nontuberculous mycobacteria (NTM), genome evolution, pulmonary infection, core genome, accessory genome, *Mycobacterium abscessus*

## Abstract

*Mycobacterium abscessus* is a multidrug resistant bacterium that causes pulmonary and extrapulmonary disease. The reported prevalence of pulmonary *M. abscessus* infections appears to be increasing in the United States (US) and around the world. In the last five years, multiple studies have utilized whole genome sequencing to investigate the genetic epidemiology of two clinically relevant subspecies, *M. abscessus* subsp. *abscessus* (MAB) and *M. abscessus* subsp. *massiliense* (MMAS). Phylogenomic comparisons of clinical isolates revealed that substantial proportions of patients have MAB and MMAS isolates that belong to genetically similar clusters also known as ‘dominant clones’. Unlike the genetic lineages of *Mycobacterium tuberculosis* that tend to be geographically clustered, the MAB and MMAS clones have been found in clinical populations from the US, Europe, Australia and South America. Moreover, the clones have been associated with worse clinical outcomes and show increased pathogenicity in macrophage and mouse models. While some have suggested that they may have spread locally and then globally through ‘indirect transmission’ within cystic fibrosis (CF) clinics, isolates of these clones have also been associated with sporadic pulmonary infections in non-CF patients and unrelated hospital-acquired soft tissue infections. *M. abscessus* has long been thought to be acquired from the environment, but the prevalence, exposure risk and environmental reservoirs of the dominant clones are currently not known. This review summarizes the genomic studies of *M. abscessus* and synthesizes the current knowledge surrounding the geographically diverse dominant clones identified from patient samples. Furthermore, it discusses the limitations of core genome comparisons for studying these genetically similar isolates and explores the breadth of accessory genome variation that has been observed to date. The combination of both core and accessory genome variation among these isolates may be the key to elucidating the origin, spread and evolution of these frequent genotypes.

## Introduction

Nontuberculous mycobacteria (NTM) include slowly and rapidly growing species in the genus *Mycobacterium* that are opportunistic pathogens in humans. A common clinical presentation is pulmonary infection, which occurs in susceptible individuals such as those with cystic fibrosis (CF), COPD, and adults with certain morphological phenotypes (Cassidy et al., 2009; Daley and Griffith, 2010; Winthrop et al., 2010; Martiniano et al., 2017). Another presentation is extrapulmonary NTM infection that can result from environmental exposures or healthcare-associated outbreaks (Lai et al., 1998; Duarte et al., 2009; Li et al., 2017). One of the most clinically challenging NTM that causes human disease is the rapidly growing species, *Mycobacterium abscessus* (*M. abscessus*), which is classified into three subspecies: *M. abscessus* subsp. *abscessus* (MAB), *M. abscessus* subsp. *massiliense* (MMAS) and *M. abscessus* subsp. *bolletii* (MBOL). MAB is the most frequently observed subspecies in clinical populations followed by MMAS, and MBOL is relatively rare (Bryant et al., 2016). Epidemiological studies in the United States (US) show an increase in pulmonary NTM infections (including *M. abscessus*) in CF populations (Olivier et al., 2003; Adjemian et al., 2018) and patients over 60 years old (Prevots et al., 2010). *M. abscessus* is also one of the most prevalent species associated with NTM infections in Europe (Roux et al., 2009; Hoefsloot et al., 2013). *M. abscessus* infections are of particular concern due to the bacteria’s innate resistance to a range of antibiotics often resulting in lengthy treatment courses and poor clinical outcomes (Jarand et al., 2011; Koh et al., 2011).

An interesting aspect of *M. abscessus* infections is that little is known about exposure risks and modes of transmission associated with pulmonary infections. The prevailing wisdom is that NTMare acquired from the environment, as they inhabit plumbing and water systems (Feazel et al., 2009; Falkinham, 2011; Ovrutsky et al., 2013; Honda et al., 2016; Zhao et al., 2017), as well as aquatic environments and soil (Primm et al., 2004; De Groote et al., 2006). These surveys have predominantly detected slowly growing NTM species, however, underscoring the potential for *M. abscessus* to occupy different and possibly yet to be identified niches within the environment. Studies of *M. abscessus* outbreaks and pseudo-outbreaks have found the offending bacteria in contaminated laboratories (Lai et al., 1998), clinic disinfectants (Tiwari et al., 2003) and hospital water sources (Baker et al., 2017), and some epidemic strains show disinfectant resistance (Duarte et al., 2009; Leao et al., 2010). Environmental surveys of NTM in household environments pinpoint the ecological niche of *M. abscessus* as indoor water and plumbing biofilms (Thomson et al., 2013a; Honda et al., 2016), *M. abscessus* also exhibits long-term survival on fomite particles (Malcolm et al., 2017). Epidemiological studies, however, have not found clear links between household exposure risks and acquisition of NTM (Prevots et al., 2014). Only two studies from Australia have demonstrated genetic links between pulmonary patient isolates of *M. abscessus* and environmental isolates from household water sources (Thomson et al., 2013a,b), though the directionality of infection has not been confirmed. Finally, attempts to isolate *M. abscessus* from environmental sources in the context of suspected outbreaks within CF centers have been unsuccessful.

The first case of person-to-person transmission of any NTM species was proposed among five CF patients with MMAS cared for at a US CF Center, raising concerns about adequacy of infection control and risks associated with patient exposure during CF care (Aitken et al., 2012). This coincided with using whole genome sequencing (WGS) to characterize genomic diversity and perform genetic strain matching. Since then, several studies have used WGS to examine genetic diversity of pulmonary *M. abscessus* isolates from patients in a US referral hospital (Davidson et al., 2014), suspected outbreaks of pulmonary infections in CF centers (Bryant et al., 2013; Tettelin et al., 2014; Harris et al., 2015; Tortoli et al., 2017); a nationwide epidemic of soft tissue infections in Brazil (Davidson et al., 2013a; Everall et al., 2017) and a global population study of CF-associated pulmonary isolates from multiple CF centers in three continents (Bryant et al., 2016). One of the most intriguing observations from these studies was that a large proportion of MAB and MMAS clinical isolates grouped into “dominant clones”, which have high genetic similarity, but are not identical. Moreover, these clones have been found in nearly every WGS study regardless of worldwide location. This finding led to speculation that the clones may have both spread locally within CF clinics, likely through ‘indirect transmission’ of contaminated clinical environments, and then globally between CF centers (Bryant et al., 2016). However, detection of these clones sporadically from non-CF patients (Davidson et al., 2014) and in cases of extrapulmonary infections (Ripoll et al., 2009; Everall et al., 2017), combined with their unknown prevalence in the environment, challenge this hypothesis.

This review will take a closer look at the genomic studies of *M. abscessus* (subspecies MAB and MMAS) and summarize observations of the dominant clones (Table 1). It will address the limitations of core genome phylogenomic analysis for genetic matching, and explore how accessory genome variation may be the key to revealing the relationships among clonal isolates from disparate geographic regions and infection types. Genomic studies of *M. abscessus* will undoubtedly be needed in the future to further study the evolution and spread of the dominant clones in both environmental and clinical settings.

**Table 1:** Isolates of the *M. abscessus* dominant clones that have been identified by WGS.

Subspecies	Infection type	Location of Study	Representative Strain(s)	Publication(s)
*M. abscessus* subsp. *massiliense* (MMAS)	CF pulmonary	Papworth Hospital, Cambridge, United Kingdom	patients 2, 8, 12, 14, 17, 19, 20, 22, 28, 29, 30	Bryant et al., 2013
	CF pulmonary	Seattle, WA, United States	Index case: 2B-0107 MAB_082312_2258 MAB_091912_2446	Aitken et al., 2012; Tettelin et al., 2014
	post surgical soft tissue	Rio de Janeiro, Brazil	CRM-0020	Duarte et al., 2009; Davidson et al., 2013a,b
	pediatric CF pulmonary	Great Ormond Street Hospital, London, United Kingdom	patient 7	Harris et al., 2015
	CF pulmonary	United States, United Kingdom, Ireland, Sweden, Netherlands, Denmark, Ireland and Australia	UNC666 (United States), SMRL275 (United Kingdom), DEN524 (Denmark), AUS755 (Australia), SVH899 (Ireland)	Bryant et al., 2016
	post surgical soft tissue	Para, Brazil	BRA_PA_42	Everall et al., 2017
*M. abscessus* subsp. *abscessus* (MAB)	post surgical soft tissue	Missouri, United States	type strain ATCC19977^T^	Moore and Frerichs, 1953; Ripoll et al., 2009
	CF and non-CF pulmonary	Colorado, United States	NJH1-6, NJH9-10	Davidson et al., 2014
	pediatric CF pulmonary	Great Ormond Street Hospital, London, United Kingdom	patients 3, 18, 19, 22	Harris et al., 2015
	CF pulmonary	United States, United Kingdom, Ireland, Sweden, Netherlands, Denmark, Ireland and Australia	UNC617 (United States), SMRL461 (United Kingdom), DEN541 (Denmark), AUS774 (Australia)	Bryant et al., 2016
	CF pulmonary	Italy	patients CP, CR, GL, BE, GM	Tortoli et al., 2017


## Genotyping Methods for *M. abscessus*

Several molecular techniques have been used to identify and classify *M. abscessus* isolates. Early studies relied on single gene sequencing or multi-locus sequence typing (MLST) using genes such as *rpoB* (Adekambi et al., 2006), *hsp65*, *secA* and the internal transcribed spacer (Adekambi et al., 2003; Adekambi and Drancourt, 2004; Zelazny et al., 2009), to classify isolates to the subspecies level. To further evaluate whether strains were “genetically matched” in the context of potential outbreaks, pulsed field gel electrophoresis (PFGE), randomly amplified polymorphic DNA polymerase chain reaction (RAPD-PCR) and repetitive sequence polymerase chain reaction (rep-PCR) (Healy et al., 2005) have been used. The highest resolution method for genetic matching is whole genome sequencing (WGS) as millions of genomic positions and thousands of loci can be compared, vs. a limited number of loci (usually less than 100) with the previous methods.

In 2009, a complete reference genome at 5.03 megabases (Mb) was published for the MAB type strain, ATCC19977^T^ (Ripoll et al., 2009) followed by genomes for the type strains of MMAS at 4.8 Mb (CCUG48898^T^) (Tettelin et al., 2012) and MBOL at 5.0 Mb (BD^T^) (Choi et al., 2012). This ushered in a new era in population genomic studies. Since 2013, several groups have utilized WGS to examine *M. abscessus* subspecies at the population level, comparing patient isolates within a single clinic (Bryant et al., 2013; Davidson et al., 2014; Harris et al., 2015) and on a larger scale, between CF clinics in one or more countries (Bryant et al., 2016; Tortoli et al., 2017).

## Genome Structure of *M. abscessus*

To understand the nuances in interpreting comparative genomics analyses, we should consider the structure of bacterial genomes, which are classified into two components. The ***core genome*** is defined as the genomic regions or genes that are shared by all isolates within a population (at the genus, species or subspecies level) (Medini et al., 2005). These include “housekeeping genes” that are necessary for survival and which evolve at relatively predictable rates. Variation in the core genome is measured by the single nucleotide polymorphisms (SNP) found in the shared regions. Depending on the species, as much as 65% or more of the genomic space can be analyzed in this way. The ***accessory genome*** includes genetic material that is shared by one or more, but not all, isolates in a population. This includes regions that are acquired by horizontal gene transfer (HGT) such as genomic islands, transposases, mycobacteriophages, and plasmids, which have the potential to change bacterial phenotypes through acquisition of antimicrobial resistance genes and entire gene cassettes that code for novel metabolic processes (Juhas et al., 2009; Sassi and Drancourt, 2014; Garcia et al., 2015; Gray and Derbyshire, 2018). Accessory genome features, such as genes, groups of genes and plasmids, are measured in an isolate population as presence or absence.

The sum of the core genes plus accessory genes in an isolate population is called the ***pan genome***. A pan genome study of 40 *M. abscessus* genomes from all three subspecies revealed a core genome of 3,345 genes, which is about 68% of a typical MAB or MBOL genome and 71% of a typical MMAS genome (Choo et al., 2014). This means that approximately 30% of the *M. abscessus* genome is made up of accessory genes that are present in only a subset of isolates or are strain-specific. The accessory genome of *M. abscessus* is known to include prophages, plasmids and genomic islands transferred by HGT from other bacterial genera (Ripoll et al., 2009; Leao et al., 2013; Davidson et al., 2014; Gray and Derbyshire, 2018) consistent with an environmental bacterium exposed to a diverse microbiome. Population genomic studies can use both core and accessory genome data in their genetic matching analyses, but it is common to find studies using only core genome comparisons as these have the most well-defined and reproducible computational methods. The accessory genome, however, can provide additional discrimination among closely related isolates.

## Genomics Studies of *M. abscessus* From CF Populations

Following the first published case of inter-patient transmission of MMAS isolates among CF patients in the US (Aitken et al., 2012), multiple European studies used core genome analysis to look for evidence of person-to-person transmission in their CF clinics (reviewed in detail in Martiniano et al., 2017). A retrospective analysis of 168 *M. abscessus* isolates from 31 adult CF patients at the Papworth hospital in the United Kingdom (UK) (Bryant et al., 2013) revealed two genetic clusters of MMAS with high genetic similarity. This study was the first to define a quantitative SNP threshold indicating probable transmission between patient isolates as less than 25 SNPs. The use of this SNP threshold for isolates from different patients coupled with social network analysis showed that while the patients lived in different geographic areas, they had multiple opportunities for cross-infection through overlapping clinic visits. Moreover, the potentially transmissible isolates shared the same SNP in the 16S rRNA that confers amikacin resistance, including isolates from patients that had not previously used nebulized aminoglycosides. The authors were unable to identify a local environmental source of MMAS inoculum, and thus surmised that person-to-person transmission did occur within their hospital. The absence of isolation in the environment, however, could also reflect insufficient culturing methods or non-exhaustive sampling. Another study at the Great Ormond Street Hospital in the United Kingdom used a similar experimental design to assess the potential for cross-infection among 20 pediatric CF patients (Harris et al., 2015). Core genome analysis of 27 *M. abscessus* isolates revealed genetic clusters of both MAB and MMAS. The authors used the suggested threshold of less than 25 SNPs between patient isolates as defined in the Papworth study (Bryant et al., 2013) and identified four patients involved in suspected transmission events, but they did not find intersecting clinic visits between any of the patients. Finally, an Italian study analyzed 162 *M. abscessus* isolates from 48 patients attending CF centers in “four geographically distinct regions in Italy” (Tortoli et al., 2017). Using a conservative threshold of less than 30 SNPs between patient isolates, they found isolate clusters of MAB, MMAS and MBOL, and identified and seven “possible transmission episodes” among patients. In three potential episodes including two clusters of MMAS and one cluster of MBOL, two patients were found to have attended the same clinic within the same timeframe. The authors concluded that the lack of major outbreaks over the 12-year study period signified minimal risk of inter-human transmission in their CF centers. In summary, the occurrence and frequency of cross-infection between CF patients in the clinic remains controversial and further studies will be needed. It would also be useful to compare and contrast infection control strategies in this context.

In 2016, a large-scale study utilized WGS and core genome analyses to examine the global population structure of 1,080 *M. abscessus* isolates from 510 CF patients sampled at clinics across multiple European countries, the US and Australia (Bryant et al., 2016). The study identified predominant clusters of genotypes that they coined “dominant circulating clones” as well as several other genetically diverse genotypes. The dominant clones are defined as phylogenetically clustered isolates found in a high proportion of patients. The study identified a primary dominant clone of MAB (known as Abscessus Cluster 1) and a dominant clone of MMAS (known as Massiliense Cluster 1) that corresponds to the “transmissible clone” from the previous Papworth study (Bryant et al., 2013). Collectively, clustered isolates had a higher proportion of drug resistance mutations to amikacin and clarithromycin and were more likely to be associated with chronic infections compared with unclustered isolates. Moreover, the clones had increased phagocytic uptake and intracellular survival in macrophages, and significantly greater bacterial burden and granulomatous inflammation in SCID mice suggesting differences in virulence compared with unclustered isolates.

## Dominant Clone of *M. abscessus* subsp. *massiliense*

Three “transmissible” MMAS isolates from the CF clinic outbreak in Seattle, Washington (Aitken et al., 2012) were sequenced in 2014, and the genomes were compared with other publically available genomes (Tettelin et al., 2014). Intriguingly, the Seattle MMAS isolates were highly similar to the transmissible MMAS clones from the Papworth, United Kingdom CF clinic study (Bryant et al., 2013) and an epidemic MMAS strain from Rio de Janiero, Brazil (CRM-0020) (Davidson et al., 2013a,b). Core genome diversity among the geographically disparate isolates was only 11 to 86 SNPs, and the accessory genome variation included a 11.5 kb genomic island that was unique to the Papworth strains and three genomic regions (totaling 95 kb) and a 44 kb IncP-1β plasmids that were unique to the Brazilian strain. Then, two years later, it was revealed that this potentially “transmissible clone” was the most prevalent MMAS (Massiliense Cluster 1) in the global population study of CF-NTM isolates and was present in all the countries sampled including the United States, United Kingdom, Ireland, Sweden, Netherlands, Denmark, Ireland and Australia (Bryant et al., 2016).

The Brazilian clone (also called BRA100) with high genetic similarity to the pulmonary CF strains was responsible for an epidemic of soft tissue infections from 2004–2009 (Duarte et al., 2009; Leao et al., 2010). The series of infections that spread through multiple states were attributed to contaminated surgical equipment that that had been cleaned with glutaraldehyde, which ultimately selected for a disinfectant resistant clone. A follow up population genomics study of 188 epidemic strains from nine Brazilian states revealed that the isolates had one or two different plasmids (pMAB01 and pMAB02) that were absent from the corresponding pulmonary CF isolates (Everall et al., 2017). Divergence dating analysis estimated that the Brazilian lineage emerged in 2003 suggesting a recent introduction, however, it is unknown if BRA100 exists locally in the environment of Brazil. The two plasmids from the Brazilian isolates have been fully sequenced (Leao et al., 2013; Everall et al., 2017), but the mechanism of glutaraldehyde resistance is still not known.

## Dominant Clone of *M. abscessus* subsp. *abscessus*

The dominant clone of MAB identified in the global NTM population study (Abscessus Cluster 1) was found in CF patients from all of the countries and continents sampled (Bryant et al., 2016). Interestingly, this clone is highly genetically similar to the MAB type strain, ATCC19977^T^, that was initially isolated in the early 1950’s from a midwestern US patient with “an acid-fast infection of the knee” and “subcutaneous abscess-like lesions of the gluteal region” (Moore and Frerichs, 1953). This clone was present in pulmonary isolate clusters found in the CF study in Great Ormand Hospital in the United Kingdom (Harris et al., 2015), the *M. abscessus* study from four Italian CF centers (Tortoli et al., 2017), and in a study of pulmonary isolates from CF and non-CF patients in a US referral hospital in Colorado (Davidson et al., 2014).

The MAB dominant clone isolates appear highly similar by core genome SNP analyses, but they also show significant variation in their accessory genomes. For example, the type strain (ATCC19977^T^) reference genome includes a 23.3 Kb plasmid with a mercury resistance operon (Ripoll et al., 2009). In the Colorado WGS study with nine clustered pulmonary isolates of the MAB dominant clone, only one (1/9 = 11%) contained the full plasmid, and up to 8.3% of the reference ATCC19977^T^ genome was absent in these isolates including a prophage region and multiple transposase-related genomic islands (Davidson et al., 2014). This study also showed that the nine isolates clustered geographically when comparing the presence or absence of genomic islands suggesting that the accessory genome may be subject to local, environmental-specific adaptations.

## Conclusion and Perspectives

The dominant clones of *M. abscessus* (including MMAS and MAB) have been observed several times over the years associated with a range of disease etiologies (Table 1), Recently, WGS and population genomics has revealed the extent of genomic variation in the core genome with less than 100 variable SNP positions, while the accessory genome can include presence or absence of large plasmids and genomic islands. The widespread geographic diversity of the dominant clones is intriguing, and it is currently not known if they have spread globally via inter-continental transmission or if they inhabitant local environments and just happen to be the most effective genotypes for human infection. A large scale genomic study of randomly sampled environmental NTM isolates would reveal the niches of *M. abscessus* dominant clones in nature and the true risk of environmental exposure to susceptible patient populations (Strong and Davidson, 2017). Such environmental studies are time consuming and costly, and appropriate funding mechanisms will need to be identified. Future genomic studies of *M. abscessus* will benefit from analyzing diversity in both the core and accessory genes to reveal local adaptations, identify potential mechanisms of virulence and monitor the evolution and mutation rates of these clinically important genotypes.

## Author Contributions

RD conceived of topic and wrote the manuscript.

## Conflict of Interest Statement

The author declares that the research was conducted in the absence of any commercial or financial relationships that could be construed as a potential conflict of interest.
